# Cornulin as a Key Diagnostic and Prognostic Biomarker in Cancers of the Squamous Epithelium

**DOI:** 10.3390/genes15091122

**Published:** 2024-08-26

**Authors:** Varun Shankavaram, Dean Shah, Aseel Alashqar, Jackson Sweeney, Hilal Arnouk

**Affiliations:** 1 Chicago College of Osteopathic Medicine, Midwestern University, Downers Grove, IL 60515, USA; 2 Public Health Program, College of Graduate Studies, Midwestern University, Downers Grove, IL 60515, USA; 3 Department of Internal Medicine, Swedish Covenant Hospital, Chicago, IL 60625, USA; 4 Department of Pathology, College of Graduate Studies, Midwestern University, Downers Grove, IL 60515, USA; 5 Precision Medicine Program, College of Graduate Studies, Midwestern University, Downers Grove, IL 60515, USA; 6 Chicago College of Optometry, Midwestern University, Downers Grove, IL 60515, USA; 7 College of Dental Medicine-Illinois, Midwestern University, Downers Grove, IL 60515, USA

**Keywords:** Cornulin, *CRNN*, squamous cell carcinoma, tumor progression, molecular pathology, prognostic biomarker, cervical cancer, head and neck cancer, esophageal cancer, skin cancer

## Abstract

The prevalence of squamous cell carcinoma is increasing, and efforts that aid in an early and accurate diagnosis are crucial to improve clinical outcomes for patients. Cornulin, a squamous epithelium-specific protein, has recently garnered attention due to its implications in the progression of squamous cell carcinoma developed in several tissues. As an epidermal differentiation marker, it is involved in skin anchoring, regulating cellular proliferation, and is a putative tumor suppressor. The physiologically healthy squamous epithelium displays a considerable level of Cornulin, whereas squamous cell carcinomas have marked downregulation, suggesting that Cornulin expression levels can be utilized for the early detection and follow-up on the progression of these types of cancer. Cornulin’s expression patterns in cervical cancer have been examined, and findings support the stepwise downregulation of Cornulin levels that accompanies the progression to neoplasia in the cervix. Additional studies documented a similar trend in expression in other types of cancer, such as cutaneous, esophageal, and oropharyngeal squamous cell carcinomas. The consistent and predictable pattern of Cornulin expression across several squamous cell carcinomas and its correlation with key clinicopathological parameters make it a reliable biomarker for assessing the transformation and progression events in the squamous epithelium, thus potentially contributing to the early detection, definitive diagnosis, and more favorable prognosis for these cancer patients.

## 1. Cornulin: Protein Structure and Biological Functions

Cornulin, also known as chromosome 1 open reading frame 10 (C1orf10), is a squamous epithelial tissue-specific protein composed of 495 amino acids, and its name is derived from the cornified layers of stratified squamous epithelia. Cornulin is encoded by the *CRNN* gene located at position 1q21.3 in the epidermal differentiation complex gene cluster, which is involved in the process of epidermal differentiation. This cluster includes three families of proteins: precursors of the cornified envelope family, S100-calcium binding proteins containing EF-hand domains, and S100-fused type proteins [1]. These proteins are essential to giving the epidermis, especially the stratum corneum, a diverse array of unique characteristics, including an immunological defensive barrier against foreign microbes, enriched lipid content for hydro-protection, and enhanced mechanical resistance and photo-protection [2]. Cornulin belongs to the S100-fused type family of proteins, which shares common structural characteristics, such as N terminus EF domains that include a 90 amino acid sequence with a calcium-binding ability. Phylogenetic analysis in mammals revealed that the *CRNN* gene might have evolved rapidly to defend against foreign pathogens, possibly as an adaptive evolutionary mechanism, through rounds of “arms races” of molecular changes by the pathogen and adaptations by the host [3] This evolutionary signature of resistance to foreign pathogens might be relevant to the pathogenesis of several cancers of the squamous epithelium, such as cervical cancer and head and neck cancers, that involve the Human Papilloma Virus (HPV) as an important etiological factor.

Cornulin is expressed mainly in squamous epithelial tissues, such as the esophagus, skin, and uterine cervix. The physiologically healthy squamous stratified epithelium has measurable Cornulin expression that is unevenly distributed among the stratified layers, with the highest expression in the superficial layers, which are also the most differentiated layers. Consequently, Cornulin is universally accepted as a differentiation marker in these tissues.

**Figure 1:**
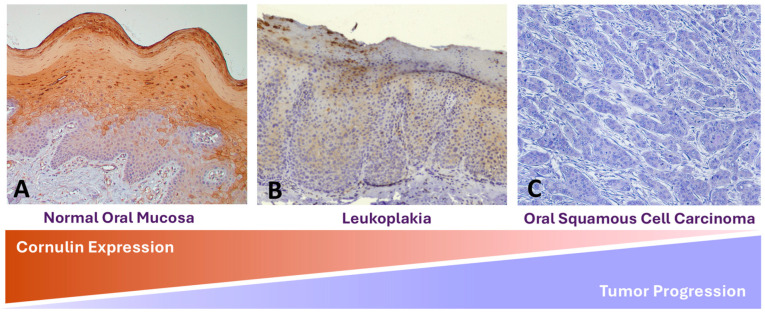
Schematic graph showing the correlation between the downregulation in Cornulin expression and the progression of oral squamous cell carcinomas from the normal oral mucosa to dysplastic premalignant lesions to invasive phenotypes. Representative immunohistochemistry staining for Cornulin in normal oral mucosa (**A**), leukoplakia lesion (**B**), and oral squamous cell carcinoma (**C**). Similar trends have been documented for cervical and esophageal cancers.

Squamous cell carcinomas, which arise in cutaneous and mucosal squamous epithelial tissues, have high morbidity and mortality rates [4]. Copious analyses of the molecular changes in squamous cell carcinomas have implicated Cornulin in the pathogenesis and progression of these malignant neoplasms. Cornulin expression seems to be downregulated in several squamous cell carcinomas, including cervical, oral, esophageal, and cutaneous squamous cell carcinoma [5,6,7,8,9,10]. Moreover, the gradual downregulation in Cornulin expression correlates with the stepwise progression to neoplasia in these lesions (Figure 1).

While the intricate molecular mechanisms of Cornulin’s role in tumorigenesis are still unknown, it has been shown to exert significant tumor-suppressive effects. Transfection of esophageal squamous cell carcinoma (ESCC) cell lines to overexpress Cornulin leads to decreased viability, proliferation, and foci-formation ability in these malignant cells. Cornulin overexpression has also been associated with tumor growth inhibition in immunocompromised mice. Indeed, the inoculation of *CRNN*-transfected ESCC cells significantly reduces tumor formation in nude mice when compared to non-transfected ESCC cells. Cell cycle analysis suggests that Cornulin arrests the cell cycle at the G1/S transition in collaboration with two other tumor suppressors, Retinoblastoma (Rb) and P21^WAF1/CIP1^. On the other hand, knockdown experiments using RNA interference to silence *CRNN* increase the proliferation and tumorigenicity of these malignant cells [11].

Cornulin, also dubbed squamous epithelial-induced stress protein of 53 kDa (SEP53), is characterized as a stress-responsive protein that can mitigate the detrimental effects of deoxycholic acid, a constituent of bile acids that can adversely impact the esophagus due to episodes of refluxed gastric acid and bile adducts [12]. During these events of deoxycholic acid-induced cell death, the upregulation of Cornulin helps limit apoptosis in the esophageal squamous epithelium. This protective effect seems to be mediated by the calcium-binding domain of SEP53.

This systematic review will focus on the expression patterns of Cornulin in several cancers of the squamous epithelium and its utility as a diagnostic and prognostic indicator of tumor progression while discussing its postulated role in tumorigenesis based on our current understanding of Cornulin’s molecular structure and biological functions. More studies are needed to shed light on the metabolic and signaling pathways that might involve Cornulin, as well as offer insights on how to utilize this knowledge to improve clinical outcomes for patients afflicted with these common types of human cancers.

## 2. Cornulin’s Expression in Different Types of Cancer

### 2.1. Cervical Cancer

Cervical cancer ranks as the fourth leading cause of cancer death in women worldwide, with an estimated number of cases of about 570,000, leading to approximately 311,000 deaths annually. Persistent infections with high-risk HPV strains, especially HPV types 16 and 18, are well-documented etiological risk factors. Squamous intraepithelial lesions, with variable degrees of dysplastic features and progression potential, might develop in HPV-infected individuals, first as low-grade squamous intraepithelial lesions (LSIL), which may progress to high-grade intraepithelial lesions (HSIL) that carry high risk for malignant transformation [13]. A cytopathological screening tool, known as the Pap test, has helped reduce the incidence of invasive cervical cancers in developed nations [14]. However, the Pap test suffers from inter-observer variability and suboptimal sensitivity and specificity, frequently leading to ambiguous findings [15]. Thus, there is an unmet need for molecular markers that enhance the accuracy of current screening protocols.

Arnouk et al. first established Cornulin as a biomarker for cervical cancer progression from its premalignant lesions in a clinical proteomics study using a combination of laser capture microdissection and two-dimensional difference gel electrophoresis (2D DIGE). Their study identified eight unique protein biomarkers that can distinguish between the normal epithelium of the cervix and the premalignant intraepithelial lesions, four that can distinguish between the premalignant intraepithelial lesions and cervical carcinoma, and sixteen that can distinguish between the normal epithelium of the cervix and cervical carcinoma. Cornulin was the only newly identified biomarker with the ability to distinguish among all these three steps in cervical cancer progression. Subsequent immunohistochemical analysis confirmed an abundant Cornulin expression in the mature upper layers of the non-keratinized squamous cervical epithelium, followed by a decline in expression in the premalignant HSIL and a further decline in invasive cervical carcinoma lesions, with undetectable levels in some cancer tissue samples [5].

Recent studies have validated Cornulin’s expression patterns in cervical cancer progression using gel-free liquid chromatography coupled mass spectrometry (LC–MS) quantitative analysis of formalin-fixed paraffin-embedded (FFPE) cervical tissues, followed by immunohistochemical detection assays. Cornulin expression showed a stepwise downregulation from the normal cervical epithelium to the squamous intraepithelial lesions (SIL) and to the cervical squamous cell carcinoma [16]. This trend was consistent with prior studies [5]. Moreover, transcriptomics studies seem to be concordant with the proteomics data regarding the downregulation in Cornulin expression as cervical cancer progresses [17]. Additionally, Cornulin expression correlated inversely with the severity of dysplasia, which indicates the risk for malignant transformation in the premalignant squamous intraepithelial lesions.

Given that the vast majority of cervical cancer cases are driven by persistent infections with high-risk Human Papilloma Virus types, especially HPV16 and HPV18 [18], and the recognized role of oxidative stress as a cancer-promoting environmental factor [19], a redox proteomics study documented the oxidative modifications of proteins in HPV-driven cervical dysplastic and neoplastic lesions. Cornulin was among a group of proteins that were more oxidized in the dysplastic lesions compared to the normal cervical epithelium [20]. Since Cornulin is less abundant in dysplastic cervical tissues [5], the oxidative damage to Cornulin is likely to further impair its functions, such as the tumor-suppressor function, in these premalignant lesions.

Recently, bioinformatics tools were employed to screen the Gene Expression Omnibus (GEO) database, containing high-throughput sequencing and microarray expression datasets, for cervical cancer biomarkers based on gene score using an artificial neural network (ANN) as a predictive computational model. Cornulin was identified among nine differentially expressed genes (DEG) that were characteristic of cervical cancer [21]. While these studies add credence to the promising diagnostic and prognostic utility of Cornulin, the exact role it plays in cervical cancer tumorigenesis is still largely unknown.

### 2.2. Head and Neck Cancer

Head and neck cancer is a general term that describes malignancies occurring in the head and neck anatomical area. Oral squamous cell carcinoma (OSCC) is the most common type of head and neck cancer; it arises from the mucosal lining of the oral cavity with increasing incidence in recent years [22]. Risk factors for the development of oral cancer include the use of tobacco and alcohol, Human Papilloma Virus (HPV) and Candida infections, genetic predisposition, exposure to harmful radiation, dietary factors, and low socioeconomic status [23]. According to the Surveillance, Epidemiology, and End Results (SEER) Program, oral cancer is associated with a relatively poor prognosis, with an overall 5-year survival rate of 69% that can significantly worsen with diagnostic delay. However, if OSCC is detected when the tumor is localized (early stages I and II), survival rates can exceed 80% [24]. Unfortunately, up to half of oral cancers are diagnosed at an advanced stage that is characterized by a large tumor size, regional lymph node involvement, or metastasis to distant organs (late stage III and IV), in which the survival rate drops down to about 40%. Moreover, survival rates for oral cancer patients have not significantly improved for decades, due largely to the challenges in early detection and the ability to identify the premalignant precursor lesions, known as leukoplakia, and determine their risk of malignant transformation [25].

The extent of OSCC growth and spread throughout the human body is typically classified using a TNM clinical staging protocol. TNM is based on the tumor size (T), lymph node involvement status (N), and metastases to distant areas (M). TNM classification plays an important role in patient management, treatment options, estimating the risk of recurrence, and the assessment of overall survival [26]. However, while TNM classification works for a group of cancer patients overall, individuals with similar TNM staging classification often have variable clinical outcomes, suggesting that reliance on TNM clinical staging alone might not be a sufficient measure for accurate prognosis. The standard treatment for OSCC involves surgery, along with radiotherapy and/or chemotherapy. Despite the combined treatment approach, approximately one-third of patients who receive surgery, combined with another therapeutic modality, experience local or regional recurrence or distant metastasis. Furthermore, patients typically experience a reduced quality of life following invasive surgery for OSCC [27].

Proteomic analysis on several human cell lines representing the successive steps in OSCC progression revealed a gradual decline in Cornulin expression as cells progressed from normal oral keratinocytes to premalignant dysplastic keratinocytes, to locally invasive OSCC, and then to metastatic OSCC cells. Specifically, Cornulin expression was reduced by about 24-fold in the locally invasive malignant keratinocytes compared to the primary gingival keratinocytes of the normal oral mucosa. Additionally, Cornulin showed more than 3-fold downregulation in dysplastic oral keratinocytes compared to normal gingival keratinocytes [8]. Several exploratory proteomics studies have confirmed the correlation between the gradual loss of Cornulin expression and the progression of oral cancer in tissue samples representing the different steps in OSCC carcinogenesis [6,28]. The possible mechanism of Cornulin downregulation in OSCC carcinogenesis was examined using DNA-sequencing techniques. Although no pathogenic mutations in the *CRNN* gene were detected, microsatellite instability (MSI)-positive OSCC tissue samples exhibited low Cornulin expression and were associated with poor prognosis [7].

Moreover, Cornulin expression was evaluated in the premalignant leukoplakia lesions in correlation with the severity of dysplasia and the potential for malignant transformation within the leukoplakia lesions. Immunohistochemical analysis of the relative Cornulin expression in high-grade oral epithelial dysplasia, low-grade oral epithelial dysplasia, normal oral mucosa, and OSCC revealed that the normal oral mucosa has the highest intensity of staining. Importantly, the staining intensity decreased as the severity of oral epithelial dysplasia increased, while OSCC samples showed the least amount of Cornulin immunoreactivity in this progression spectrum. Moreover, the well-differentiated OSCC (histopathological grade G1) exhibited more intense staining compared to the poorly differentiated OSCC (histopathological grades G2 and G3), consistent with the role of Cornulin as a differentiation marker [29].

Since the tongue is the most common anatomical site for OSCC, a study that focused on the expression of Cornulin in tongue squamous cell carcinoma (TSCC) demonstrated high Cornulin expression in non-malignant oral tissues and a reduced expression in TSCC tissues. Moreover, the reduced Cornulin expression was associated with the loss of differentiation, designated as high histopathological grades (G2, G3), and with lymph node involvement in TSCC. The study also correlated low Cornulin expression with the largest tumor dimension [30].

In laryngeal squamous cell carcinoma (LSCC), a subtype of head and neck cancer, Cornulin was identified as a critical hub connecting several differentially expressed genes in LSCC using bioinformatics tools to screen for differentially expressed genes in the Gene Expression Omnibus (GEO) genomics data repository. Additionally, Cornulin downregulation in LSCC was documented at the gene and protein levels of expression and found to be associated with poor clinical outcomes using Kaplan–Meier plots for survival rate analysis. Moreover, the overexpression of Cornulin in LSCC cell lines inhibited their growth and their progression through the cell cycle, attenuated their migration and invasion, and inhibited their tumor formation when implanted subcutaneously into nude mice [31].

**Figure 2:**
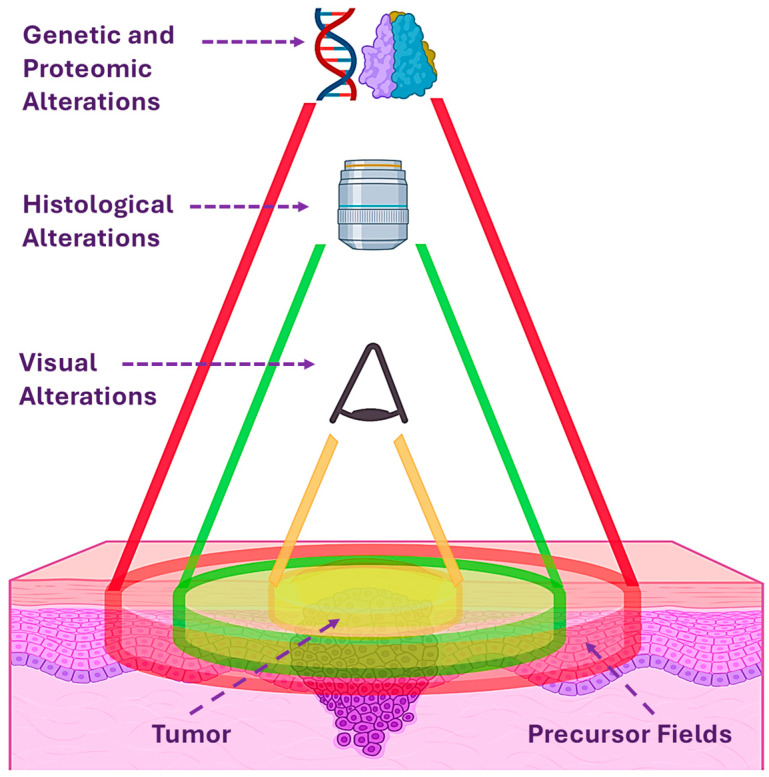
Illustration of evaluating the extent of tumor spread and margins using direct visual examination of tumor mass (yellow zone), microscopic examination of histological alterations (green zone), and molecular studies to reveal genetic and proteomic alterations in the precursor fields (red zone) that can suffer malignant transformation leading to local relapses in head and neck cancer patients.

Cornulin also proved to be a potential predictor of local relapse, which is a major clinical challenge, with an estimated 20% to 40% of oral cancer patients suffering from a local relapse in the vicinity of the resected tumor despite showing cancer-free surgical margins [32,33]. This is likely due to the presence of patches of genetically altered keratinocytes, known as precursor fields, that might not be visually detectable and might not be completely resected with the primary tumor. Local relapses are thought to be the result of malignant transformation in these precursor fields (Figure 2) [34,35].

Importantly, the detection of Cornulin expression in non-involved mucosal surgical margins of OSCC tumors may serve as an independent indicator for predicting the local relapse of oral squamous cell carcinoma. Studies utilizing immunohistochemistry staining of surgical margins of patients’ tissue samples from a relapse group, non-relapse group, and normal oral mucosa showed that a significant decrease in Cornulin expression is associated with local relapse cases. It is worth mentioning that the patient’s age was also an independent predictor of relapse in OSCC patients [36]. In a separate study, Cornulin and Keratin 4 were selected from a group of protein biomarkers that are differentially expressed between normal and cancer tissues, and their expression was examined in the surgical margins of 46 patients with head and neck squamous cell carcinoma. The subset of patients with the aberrant expression pattern of low Cornulin and low Keratin 4 suffered more local relapses and had worse survival rates. Interestingly, the expression of both Cornulin and Keratin 4 in the surgical margins of resected tumors predicted local relapse better than the traditional histopathological grading of dysplasia. This could be explained by the reproducibility and objectivity of immune-detection methods of protein biomarkers versus the variability and subjectivity associated with the morphology-based manual grading of dysplasia [6].

Recent studies have identified salivary Cornulin as a potential biomarker for oral cancer screening. The saliva offers a medium suitable for cancer screening in a convenient, non-invasive, and pain-free collection procedure, although confounding variables, such as fasting, diet, medications, and overall health/disease status, should all be considered in order to standardize saliva collection from participants [37,38]. A liquid chromatography–mass spectrometry proteomic analysis on saliva samples obtained from oral cancer patients and healthy control individuals identified Cornulin among six differentially expressed proteins that can discriminate between the two compared groups. Specifically, salivary Cornulin levels were significantly lower in oral cancer patients compared to non-cancer subjects [39]. These findings were confirmed using Western blotting detection methods on saliva samples from the compared groups [28]. Overall, salivary Cornulin consistently and significantly displayed high sensitivity and specificity as a biomarker for oral cancer screening. Interestingly, circulating Cornulin levels in the plasma showed a strong linear dose-dependent correlation with the use of Swedish moist snuff (snus), a suspected risk factor for cancer development [40]. Separately, Cornulin expression significantly correlated with the use of the addictive agent Naswar, a smokeless tobacco product, in oral cancer tissues [30]. Based on all these findings, we can postulate a protective role for Cornulin in the oral mucosa in response to DNA-damaging risk factors, such as tobacco and alcohol use, and a mechanism by which Cornulin downregulation allows for these malignant tumors to occur and progress to advanced stages.

### 2.3. Esophageal Cancer

Esophageal cancer is a major global health challenge, ranking eighth globally in prevalence and standing as the sixth leading cause of cancer-related deaths. It primarily exists in two forms: esophageal squamous cell carcinoma development (ESCC) and esophageal adenocarcinoma. The two subtypes of esophageal cancer exhibit significant geographical differences. Populations in developing countries with high rates of environmental risk factors, such as alcohol and tobacco use, poor oral hygiene, and low socioeconomic status, are most at risk for ESCC [41]. Conversely, incidences of esophageal adenocarcinoma are higher in developed countries, largely due to the prevalence of Barrett’s Esophagus, a condition caused by chronic gastroesophageal reflux disease (GERD) [42]. In both types of esophageal cancer, genetic mutations in oncogenes or tumor suppressor genes are believed to accumulate due to damage to the esophageal tissue, leading to tumor growth [9]. ESCC is found to be the most common subtype worldwide, and despite its significant incidence, the five-year survival rate remains low at around 21%, largely due to inadequate screening procedures and late-stage diagnosis, often post-metastasis [42]. After metastasis, the five-year survival rate drops to a mere 5% based on recent data from the National Cancer Institute. These statistics highlight the demand for novel biomarkers to aid in early disease detection or serve as targets for therapeutic interventions.

Studies have shown a differential gene expression between normal esophageal epithelial cells and ESCC cells [43,44]. *CRNN*, the gene encoding for Cornulin, was amongst the newly identified differentially expressed genes with significant downregulation in ESCC compared to normal esophageal cells. The malignant ESCC cells exhibited a 247-fold downregulation compared to normal differentiated squamous epithelium [45]. Another study has validated the decrease in Cornulin levels of ESCC cells using immunohistochemical staining. In 89% of the ESCC tissue samples, no Cornulin was observed. In the remaining 11%, slight Cornulin levels were observed in the cytoplasm of early stage ESCC cells, suggesting that Cornulin levels continue to decrease as esophageal cancer progresses [9]. Quantitative analysis has also demonstrated the differential expression of this gene. Using a novel technology that increases the precision of tissue procurement, expression microdissection (xMD) was used to obtain ESCC samples that would be optimal for protein analysis. The results highlighted the significant reduction in Cornulin in ESCC cells when compared to the normal epithelial tissues of the esophagus. In fact, Cornulin was not found to be expressed in any tumor sample examined, validating previous studies documenting a reduced expression of *CRNN* in esophageal carcinoma tissue [46].

Moreover, the clinical implications of Cornulin levels are substantial. The downregulation of *CRNN* is directly correlated with advanced clinical stages of ESCC and lymph node metastasis. More aggressive forms of ESCC have markedly lower expression levels of Cornulin than earlier stages [11]. Studies have shown that *CRNN* expression is correlated with tumor invasion depth and TNM clinical staging. Patients with negative *CRNN* expression had a greater tumor length and more lymph node involvement than those with positive *CRNN* expression, suggesting the role this protein has in tumorigenesis [47]. Additionally, patients with decreased *CRNN* expression levels experienced an average survival time of 36 months compared to an average survival time of 51 months for those with normal gene expression levels [11]. Another study found that patients with positive *CRNN* expression had a median survival of 30 months and a five-year survival of about 40%, while those with negative *CRNN* expression had a median survival of 14 months and a five-year survival of approximately 20% [47]. Using multivariate analysis, both studies found that *CRNN* downregulation can be used as an independent prognostic predictor for ESCC, highlighting its strong correlation to clinical outcomes [11,47]. At the molecular level, ESCC cell lines transfected to overexpress Cornulin exhibited reduced viability and growth using in vitro and in vivo models, consistent with the proposed tumor-suppressor role of Cornulin, while silencing *CRNN* with RNA interference resulted in an increase in the tumorigenicity of these cells [11].

While most studies emphasize the differences between normal esophageal epithelial tissue (NE) and invasive esophageal squamous cell carcinoma, recent scientific investigations have focused on the precursor lesions, known as esophageal squamous precancerous lesions (ESPL). ESPLs are generally categorized into two types: low-grade intraepithelial neoplasia (LGIN) and high-grade intraepithelial neoplasia (HGIN), which is characterized by progressive dysplasia. Few studies have included ESPL lesions due to the challenge of obtaining sufficient tissue from the small areas involved. A recently developed technique, called spatial transcriptome analysis, has allowed for a precise selection of ESPL areas in the tissue while minimizing interference from surrounding areas, which revealed several differentially expressed genes across the progression spectrum of esophageal cancer. *CRNN*, in particular, exhibited a consistent decrease in relative expression, with the most significant decline occurring from NE to LGIN and from LGIN to HGIN, while its expression remained constant from HGIN to ESCC, suggesting its major regulatory role in ESCC progression [48]. Similarly, immunohistochemical analysis reflected a similar trend, with ESCC showing an even lower score when compared to HGIN. Subsequent mechanistic studies confirmed that the overexpression of Cornulin in ESCC cell lines inhibits cellular proliferation and tumor formation in patient-derived xenograft models (PDX) [48]. Altogether, these findings validated previous studies about the role of Cornulin in the development of esophageal cancer.

Ultimately, esophageal cancer remains a significant global health issue, highlighting the need to find better screening procedures and novel biomarkers that can aid in earlier detection and better treatment. The emerging evidence of Cornulin’s role as a potential diagnostic and prognostic biomarker in esophageal cancer offers promising clinical utility.

### 2.4. Skin Cancer

Cornulin has been found to play a role in a common type of skin cancer known as cutaneous squamous cell carcinoma (cSCC), which is one of three major types of malignancies in the skin and is the second most deadly after melanoma and the second most common after basal cell carcinoma [49]. While the overall rate for metastatic disease is about 14%, the metastasis rate in patients with high-risk features is 37%, and the mortality rate of metastatic cSCC is 70% [50,51,52,53,54,55]. High-risk features for metastatic cSCC include a large tumor size; immunocompromization; the depth of invasion; poor differentiation; lymphovascular or perineural involvement; or localization of the lesion to the ears, lips, or genitals [56]. Like many cancers, metastatic cSCC has the potential to recur. Its recurrence rate ranges from 15% to 28% [57,58]. These data suggest that the early diagnosis of primary cSCC tumors and their metastases, followed by the appropriate treatment, is crucial for patient survival and wellbeing.

The incidence of new cutaneous squamous cell carcinoma lesions has risen in recent years. This is likely due to increased ultraviolet radiation exposure due to sunbathing and tanning bed use and low use of sun protection factor (SPF) sunscreen throughout the past century, in addition to occupational exposure and weakening of the ozone layer due to climate change [59,60,61]. Interestingly, Black Americans have a higher mortality rate from cSCC compared to White Americans due to the majority of cSCC diagnosed in Black Americans being found in non-sun-exposed skin, where it has a greater potential for metastasis and gets diagnosed at later stages [62,63].

**Figure 3:**
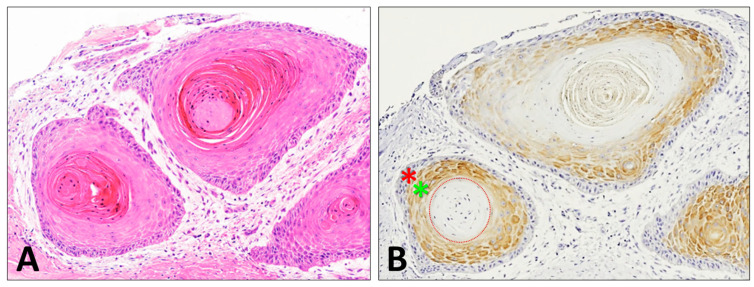
Cornulin expression around keratin pearls in well-differentiated cutaneous squamous cell carcinoma tissue samples. Representative images (**A**) H&E-stained and (**B**) Immunohistochemistry-stained show intense Cornulin immunoreactivity in the central keratinocytes (green asterisk) adjacent to the keratin pearl (dotted circle), while the peripheral keratinocytes (red asterisk) do not show any detectable levels of Cornulin expression.

Currently, the diagnosis of cSCC is limited to histopathologic examination based on morphology, and there are no established biomarkers to diagnose or prognosticate cSCC. Cornulin has shown great potential to serve as a biomarker for detecting cSCC. As the epidermal tissue differentiates, Cornulin expression increases, specifically in the distal epidermal layers [64]. There is about a 2-fold decrease of Cornulin expression in cutaneous squamous cell carcinoma compared to the normal epidermis [10]. Notably, Cornulin expression decreases within cSCC as the tumor histopathological grade increases. Approximately a 4.5-fold decrease in Cornulin expression has been documented in high-grade cSCC (G2 and G3) compared to low-grade (G1) cSCC tissue samples, which means that poorly differentiated cSCC tumors express even less Cornulin than well-differentiated tumors [10]. The accurate determination of the tumor differentiation status is a useful prognostic indicator because differentiation inversely correlates with the aggressive biological behavior of malignant neoplasms [65]. These findings resulted from a blinded study using immunohistochemistry (IHC) staining and computer-assisted image analysis, and immunoreactivity was determined by calculating a Histo-score (H-score) that factored in the percentage of positively stained epithelial cells and the intensity of staining. This method was able to objectively estimate the relative amount of Cornulin present in keratinocytes instead of relying solely on morphology-driven examination, which can suffer from inter-observer variability [10]. Interestingly, Cornulin staining is intense in cSCC cells that are adjacent to the keratin pearls found in well-differentiated cSCC, while cSCC cells that are not directly adjacent to the keratin pearls show no detectable levels of Cornulin (Figure 3).

A recent study analyzed the tumor interstitial fluid of basal and squamous cell carcinoma and found that while there was an increase in Cornulin protein in the interstitial fluid of basal cell carcinoma cells, there was a decrease in Cornulin in the interstitial fluid of squamous cell carcinoma cells [66].

Given that cSCC can metastasize or recur, it is imperative during diagnosis to determine if there has been any metastasis. TNM staging is performed to determine the size of the tumor, regional lymph node involvement, and distal metastasis when cancer is first diagnosed. This specific cancer tends to metastasize first to regional lymph nodes before traveling to distal tissues, and mortality is often driven by regional metastasis compared to distal metastasis [67,68]. Karumuri et al. used immunohistochemical analysis to quantitatively show a significant 9.5-fold downregulation in Cornulin expression in primary cSCC tumors with N1 Status compared to primary cSCC tumors with N0 Status [69]. These findings have clinical implications since occult metastasis, often referred to as micrometastasis, can occur and may not be detected by morphology-driven diagnostic methods. This can lead to false negatives and affect clinical outcomes. The detection of Cornulin in primary tumors may offer a reliable test to predict the presence of occult metastases that evade detection by traditional methods.

The research on Cornulin’s involvement in cSCC is currently limited, and there are no current studies correlating Cornulin’s expression to survival rates, relapse, or use in current protocols to diagnose cSCC. Using immunohistochemistry to quantify the amount of Cornulin in both suspected cSCC samples and regional lymph nodes could assist with the current morphology-driven diagnostic methods and aid in detecting both primary lesions and micrometastasis, thus improving clinical outcomes and survival rates for these patients.

## 3. Conclusions

In recent years, there has been mounting evidence for Cornulin’s association with squamous cell carcinomas of the cervix, esophagus, head and neck, and skin. Specifically, a significant downregulation in Cornulin expression correlates with several important clinicopathological parameters, individually or combined, such as the differentiation status, lymph node involvement, local relapse, advanced clinical stages, and/or reduced survival rates. Moreover, Cornulin expression inversely correlates with the severity of dysplastic changes in the premalignant lesions that precede these invasive carcinomas. Thus, Cornulin may also aid in the risk stratification of malignant transformation in these precursor lesions. However, since Cornulin is progressively downregulated in premalignant lesions and squamous cell carcinomas, especially the poorly differentiated phenotypes, it might be necessary to resort to highly sensitive detection methods, along with the appropriate control specimens, to ensure reliable measurements before it can be utilized in cancer screening and early detection.

Altogether, Cornulin holds great promise as a molecular biomarker that can objectively and accurately diagnose several types of cancers, determine the malignant potential of precursor lesions, predict clinical outcomes, monitor disease progression and recurrence, and ultimately help guide treatment options for patients afflicted with these malignancies.

## 4. Future Perspectives

While several proteomics, transcriptomics, and bioinformatics studies have identified Cornulin as the “Passepartout”, or master key, prognosticator for cancers of the squamous epithelium, clinical follow-up studies are needed to help establish this promising biomarker as a standard test that can complement the morphological pathology examination of squamous cell carcinomas. Additionally, molecular mechanistic investigations into the biological role of Cornulin can provide valuable insights into the broader pathogenesis and etiology of these cancers.
